# A novel *in vitro* cyclic micropropagation protocol and assessment of genetic fidelity in the critically endangered woody species *Carpinus putoensis*

**DOI:** 10.3389/fpls.2026.1791775

**Published:** 2026-03-11

**Authors:** Peidong Chen, Wulin Zheng, Yumei Xie, Yuan Chen, Binjie Ge, Yonghong Hu, Mulan Zhu

**Affiliations:** 1 National Key Laboratory of Plant Molecular Genetics (NKLPMG), Chinese Academy of Sciences (CAS) Center for Excellence in Molecular Plant Sciences, Chinese Academy of Sciences, Shanghai, China; 2 Shanghai Key Laboratory of Plant Functional Genomics and Resources, Shanghai Chenshan Botanical Garden, Shanghai, China; 3 Department of Social Cooperation and Development (School of Rural Revitalization), Wuhan Technology and Business University, Wuhan, China

**Keywords:** *Carpinus putoensis*, cyclic organogenesis, endangered species, genetic stability, heterologous micrografting, woody tree species

## Abstract

*Carpinus putoensis* is a critically endangered woody tree species with only a single known wild individual, facing severe reproductive barriers. To overcome the scarcity of germplasm, this study established a stable “*in vitro*–ex vivo–*in vitro*” cyclic micropropagation system. The protocol consists of three key stages. Initially (Primary Culture Establishment), semi-lignified stem segments were collected as initial explants from mature *C. putoensis* trees (derived from cuttings of the single wild individual). In this phase, the axillary bud induction rate was 28.6%, but the shoots exhibited slow elongation and severe rooting recalcitrance. To rescue this valuable germplasm, a modified heterologous micrografting technique was employed using *in vitro C. putoensis* shoots as scions and *Glycine max* (soybean) seedlings as rootstocks. Subsequently (Ex Vitro Acclimatization), the micrografted plantlets were successfully transferred to a greenhouse, serving as the source of explants for the next phase. Finally (*In Vitro* Culture Re-establishment), nodal segments from the acclimatized mother plants were re-introduced into culture. In this sustainable phase, shoot proliferation was significantly improved on WPM medium containing 0.3 mg·L^-^¹ 6-BA, 0.03 mg·L^-^¹ NAA, and 2 g·L^-^¹ activated charcoal, achieving a high axillary bud induction rate of 90.31%. A mean shoot elongation of 4.53 cm was obtained using 0.1 mg·L^-^¹ 6-BA and 0.8 mg·L^-^¹ GA_3_, followed by a high rooting rate (89.3%) using a two-step IBA treatment. Genetic analysis using RAPD and ISSR markers confirmed the genetic fidelity of the micropropagated plantlets. This cyclic system provides a renewable and genetically stable method for the conservation of *C. putoensis*.

## 1 Introduction

*Carpinus putoensis* W. C. Cheng (Betulaceae), first taxonomically identified in 1930 (Cheng, 1932), is recognized as one of the most critically endangered plant species worldwide (Li et al., 2010; Qin and Zhao, 2017; Sheng et al., 2021a). With only a single wild mother tree remaining on Putuo Mountain, China (Wade et al., 2016; Sheng and Zhu, 2018), it is listed as a Grade I National Key Protected Wild Plant (Meng et al., 2004) and categorized as Critically Endangered (CR) on the IUCN Red List (IUCN, 2024). Consequently, effective conservation strategies are urgently needed to rescue this species from extinction.

The critical status of *C. putoensis* stems from both habitat degradation (Li et al., 2025b) and severe reproductive barriers. Sexual reproduction is restricted by pronounced dichogamy and low pollen viability, resulting in a viable seed rate of only 2–4% (Lu and Zou, 1990). While *ex situ* conservation efforts have successfully established flowering populations (Zhao et al., 2020), these populations remain vulnerable to genetic erosion and lack the co-evolutionary pressures of their native habitat (Exposito-Alonso et al., 2022). Similarly, vegetative propagation via cuttings is constrained by strict seasonal windows and the physiological state of source materials (Singh et al., 2011; Li et al., 2025a). The scarcity of propagules from the single mother tree (Ma et al., 2013), combined with long reproductive cycles (Li et al., 2010), results in a critically low propagation coefficient. Thus, relying solely on traditional approaches is insufficient for rapid population recovery, highlighting the urgent need for highly efficient biotechnological alternatives.

Plant tissue culture (micropropagation) serves as a powerful solution for rapid, large-scale clonal propagation (Sarasan et al., 2006; Thorpe, 2007), effectively circumventing challenges such as low seed viability and seasonal restrictions (Kulak et al., 2022). Robust micropropagation protocols have been established for other endangered woody plants, including *Betula oycoviensis* (Vitamvas et al., 2020) and *Acer glabrum* var. *douglasii* (Hathaway et al., 2020). Furthermore, advanced *in vitro* technologies have been instrumental in the conservation of threatened trees like *Prunus africana* (Komakech et al., 2020) and *Magnolia sirindhorniae* (Cui et al., 2019). To ensure clonal fidelity, molecular marker techniques such as Random Amplified Polymorphic DNA (RAPD) and Inter-Simple Sequence Repeat (ISSR) are widely employed to detect potential somaclonal variation (Bairu et al., 2010; Krishna et al., 2016). Despite these successes, a comprehensive micropropagation system for *C. putoensis* that systematically validates genetic fidelity throughout the “*in vitro*–ex vivo–*in vitro*” cycle has yet to be reported.

This study establishes the first complete, efficient, and cyclic micropropagation system for *C. putoensis* with confirmed genetic fidelity. By optimizing explant sterilization, overcoming rooting obstacles via heterologous Hypocotyl-Apical Bud (HAB) micrografting, and establishing an efficient re-established culture cycle—including optimized axillary bud induction and a two-step rooting method—this system effectively addresses the bottleneck of low propagation efficiency. Validated by 100% genetic fidelity using molecular markers, this work provides a vital technical platform for the *ex situ* conservation of *C. putoensis* and offers a methodological reference for other recalcitrant woody species.

## 2 Materials and methods

### 2.1 Plant resource

**Figure 1:**
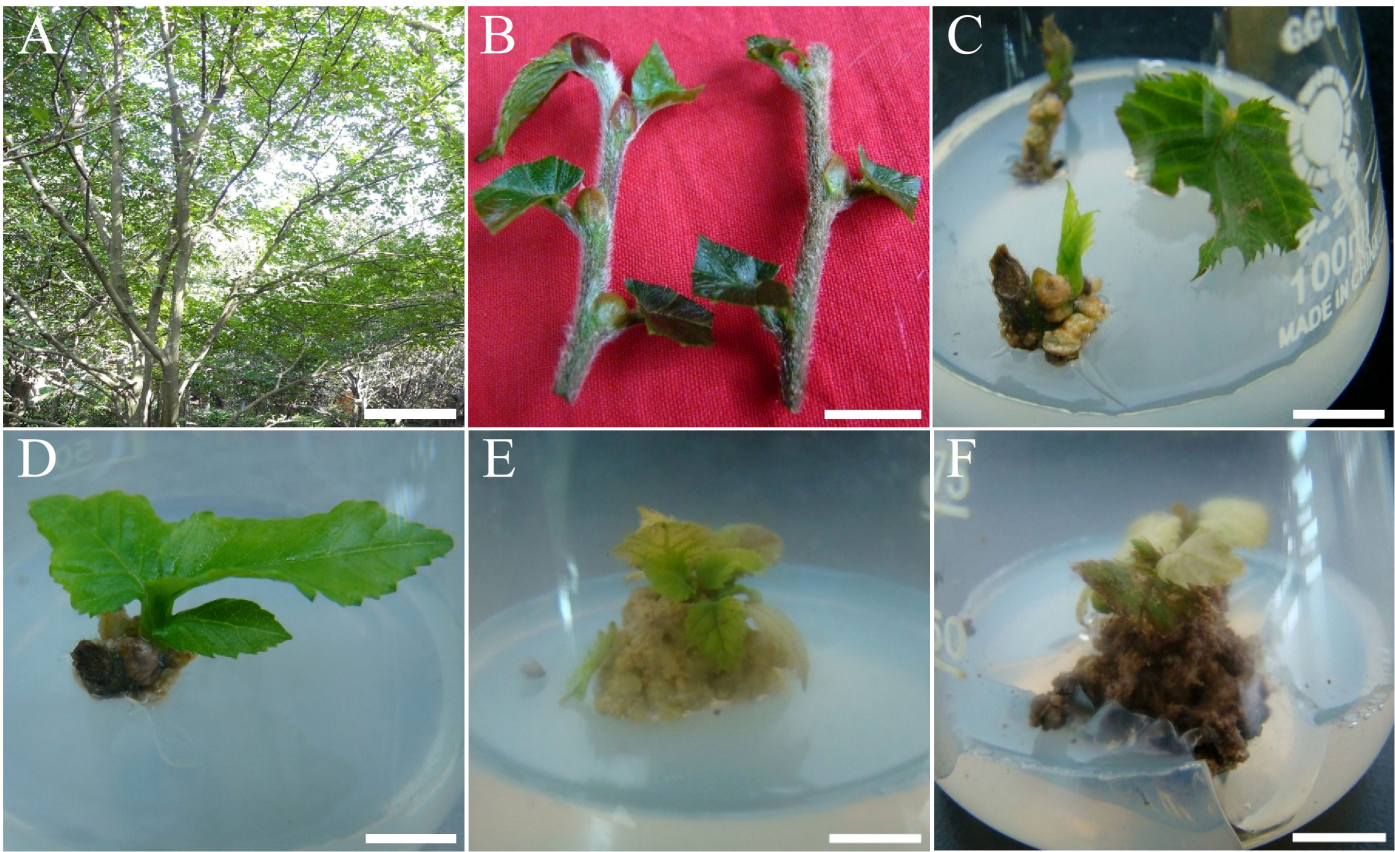
Primary culture and response of *C. putoensis* explants to different hormone concentrations. **(A)** Plants derived from cuttings of the wild mother tree in Zhoushan, Zhejiang. **(B)** Stem segment explants prepared for surface sterilization. **(C)** Axillary bud elongation after 4 weeks on hormone-free MS medium. **(D)** Axillary bud sprouting after 4 weeks on MS medium with 0.5 mg·L^-^¹ 6-BA and 0.05 mg·L^-^¹ NAA. **(E)** Callus formation and suppressed shoot growth after 4 weeks on MS medium with 1.0 mg·L^-^¹ 6-BA and 0.2 mg·L^-^¹ NAA. **(F)** Callus browning and necrosis after 4 weeks on MS medium with 3.0 mg·L^-^¹ 6-BA and 0.5 mg·L^-^¹ NAA. Scale bars: **(A)** = 2.6 m; **(B)** = 1.3 cm; **(C–E)** = 1 cm; **(F)** = 1.2 cm.

Initial explants (stem segments) were collected from plants derived from cuttings of the wild mother tree of *C. putoensis* located on Putuo Mountain, Zhoushan, Zhejiang Province, China (approx. 30.015° N, 122.385° E) (**Figure 1A**). The taxonomic identity of the source material was confirmed based on morphological characteristics described in *Flora of China* (Li and Skvortsov, 1999).

### 2.2 Culture medium and conditions

The basal media used in this study were Murashige and Skoog (MS) medium (Murashige and Skoog, 1962) and Woody Plant Medium (WPM) (Lloyd and McCown, 1980). Unless otherwise specified, all media consisted of their respective salts and vitamins, were supplemented with 30 g·L^-^¹ sucrose and solidified with 5 g·L^-^¹ agar (gel strength: 1300 g·cm^-^²). The pH of the media was adjusted to 5.8 before being autoclaved at 121 °C and 0.105 MPa for 20 min. All cultures were incubated at 25 ± 2 °C in a growth chamber under a 16 h/8 h (light/dark) photoperiod. Illumination was provided by cool-white fluorescent lamps at a photosynthetic photon flux density (PPFD) of 33.6 µmol·m^-^²·s^-^¹. All basal medium components, sucrose, and agar were purchased from Hangzhou Lin’an Pingzhuang Scientific Experiment Supplies (Hangzhou, China). All plant growth regulators (PGRs) were purchased from Hangzhou Maibo Biotechnology Co., Ltd. (Hangzhou, China), and Plant Preservative Mixture (PPM) was purchased from Shanghai Yeasen Biotechnology Co., Ltd. (Shanghai, China).

### 2.3 Preparation of sterile explants

To establish the “*in vitro*–ex vivo–*in vitro*” cyclic micropropagation system, two types of source materials were used in this study. Explants collected from plants derived from cuttings of the wild mother tree for Primary Culture Establishment are hereinafter referred to as primary explants. Explants collected from 2.5-year-old acclimatized plantlets (growing in the greenhouse) for *In Vitro* Culture Re-establishment are referred to as re-initiated explants.

The source material for obtaining primary explants was healthy, current-year semi-lignified stem segments collected from plants derived from cuttings of the wild mother tree (**Figure 1B**). To remove dense surface trichomes, stems were briefly immersed in 95% (v/v) ethanol for approximately 1 s and then rapidly passed through the flame of an alcohol lamp. The treated stems were cut into 3–5 cm segments and rinsed thoroughly under running tap water for 2 h. Subsequently, they were immersed in a 0.5% (w/v) potassium permanganate solution (0.5 g KMnO_4_ dissolved in 100 mL distilled water) for 30 min, with ultrasonic cleaning applied during the first 15 min. After being blotted dry with sterile filter paper, the explants were transferred to a laminar flow hood for surface sterilization. They were immersed in 75% (v/v) ethanol for 50 s, followed by treatment with a mixed sterilizing solution containing 1% (v/v) benzalkonium bromide and 50% (v/v) Plant Preservative Mixture (PPM) for 7 min. Finally, the explants were rinsed five times with sterile distilled water.

**Figure 2:**
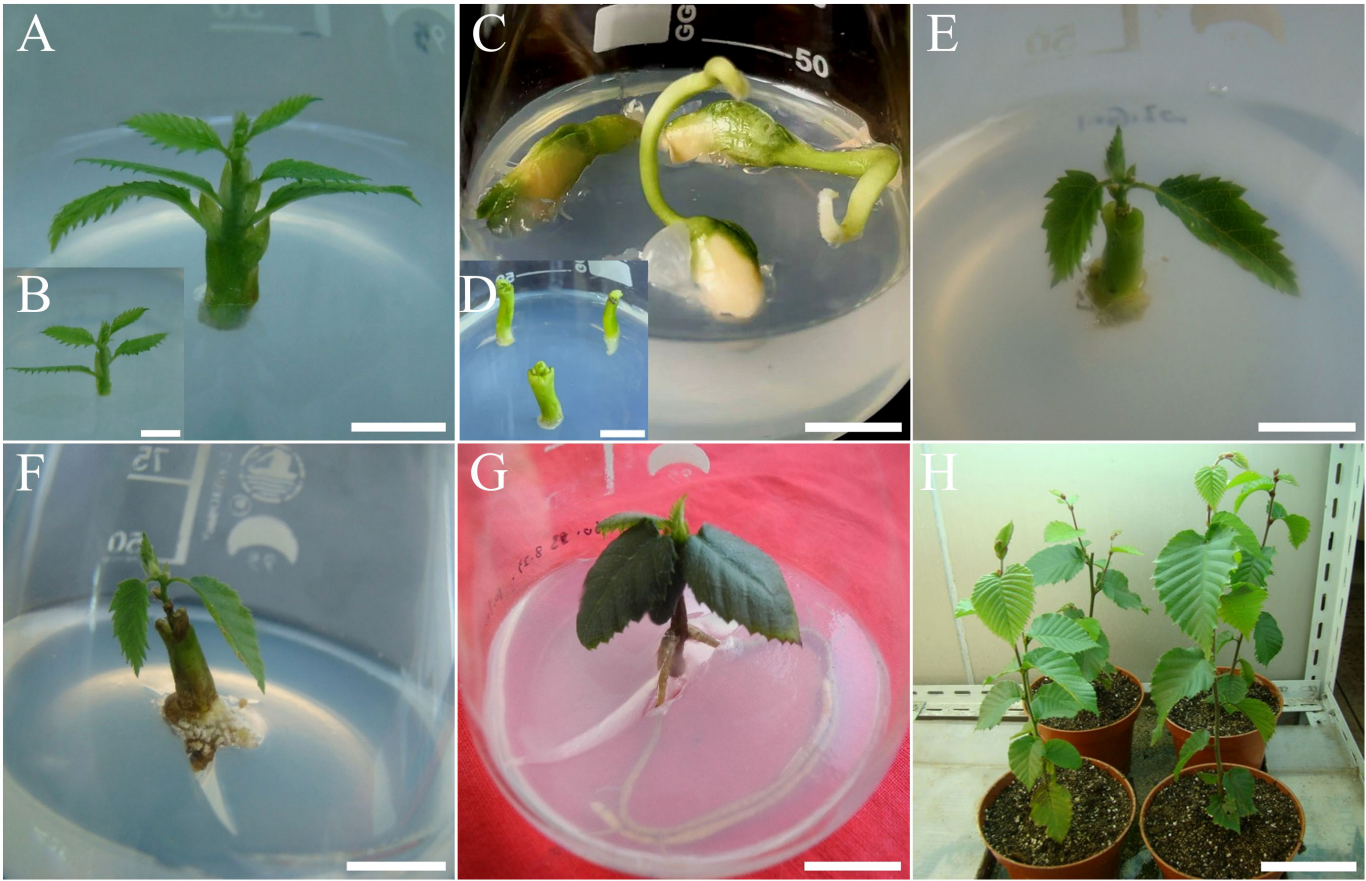
*In vitro* rooting of *C. putoensis* shoots using a soybean hypocotyl-apical bud (HAB) micrografting system and subsequent acclimatization. **(A)** Shoot selected for rooting experiments. **(B)** The same shoot after being trimmed. **(C)** Soybean seeds germinated for 1 week on MS medium containing 3.0 mg·L^-^¹ 6-BA. **(D)** Soybean hypocotyls excised from seedlings and cultured for 1 week on hormone-free MS medium. **(E, F)** Shoots undergoing root induction pre-treatment via micrografting with soybean hypocotyls for 5 days **(E)** and 10 days **(F)** on MS medium containing 0.1 mg·L^-^¹ 6-BA and 1.0 mg·L^-^¹ IBA. **(G)** Plantlet after 7 weeks of subsequent culture on WPM medium supplemented with 1.0 mg·L^-^¹ IBA. **(H)** Acclimatized plantlets after 2.5 years of growth under ex vitro conditions. Scale bars: **(A, C)** = 1.3 cm; **(B)** = 1 cm; **(D)** = 0.9 cm; **(E)** = 1.5 cm; **(F, G)** = 1.2 cm; **(H)** = 14 cm.

The source material for obtaining re-initiated explants was stem segments collected from the 2.5-year-old acclimatized plantlets (**Figure 2G**). The preparation began with a pre-cleaning step: the surfaces were wiped with cotton moistened with 75% (v/v) ethanol. These explants were then cut into segments and rinsed under running tap water for 2 h. After blotting dry with sterile filter paper, a series of treatments were tested to optimize the surface sterilization protocol within a laminar flow hood. Explants were first immersed in 75% (v/v) ethanol for two different durations (30 and 50 s). Subsequently, they were treated with a mixed sterilizing solution containing 1% (v/v) benzalkonium bromide and varying concentrations of PPM (20% and 50%, v/v) for three different durations (5, 7, and 9 min). After sterilization, explants were rinsed five times with sterile distilled water and inoculated onto hormone-free MS medium. Each treatment consisted of 20 replicates, and contamination and survival rates were recorded after 2 weeks of culture.

### 2.4 Bud induction culture of primary explants

The primary explants (prepared as described in Section 2.3) were inoculated onto MS basal medium to screen for optimal induction conditions. Four treatments were established by combining 6-BA at concentrations of 0, 0.5, 1.0, and 3.0 mg·L^-^¹ with NAA at 0, 0.05, 0.2, and 0.5 mg·L^-^¹, respectively.

Each culture vessel (100 mL Erlenmeyer flask) contained 50 mL of medium. The experiment followed a completely randomized design with 7 replicates per treatment, and each replicate consisted of one explant per vessel. The total culture period was 4 weeks. To prevent browning and nutrient depletion, the explants were transferred to fresh medium of the same composition every 2 weeks. At the end of the 4-week period, the axillary bud sprouting rate (%) and callus formation rate (%) were recorded. Additionally, the morphological status of the explants was observed and described.

### 2.5 Micrografting for rooting and acclimatization of primary shoots

To induce adventitious rooting in recalcitrant primary shoots, a heterologous Hypocotyl-Apical Bud (HAB) micrografting technique was employed. Primary shoots (*C. putoensis*) obtained from the primary culture served as the apical bud scions. Rootstocks were prepared from the hypocotyls of soybean (cv. ‘Jiaoda-18’) seedlings. This cultivar was specifically selected as the nurse rootstock because preliminary trials indicated that its hypocotyls are sufficiently stout to support the scion and rich in endogenous hormones, which facilitates the rejuvenation and rooting of recalcitrant woody species. Each culture vessel (100 mL Erlenmeyer flask) contained 50 mL of medium.

First, soybean seeds were germinated for 1 week on MS medium containing 3.0 mg·L^-^¹ 6-BA. Subsequently, the hypocotyls were excised from the seedlings and cultured on hormone-free MS medium for another 1 week. The prepared rootstocks were then decapitated, and a longitudinal slit was made in the hypocotyl. The scions were inserted into the slit and co-cultured for 2 weeks on MS medium with 0.1 mg·L^-^¹ 6-BA and 1.0 mg·L^-^¹ IBA. Following this HAB co-culture phase, the scions were excised from the rootstocks and transferred to WPM medium supplemented with 1.0 mg·L^-^¹ IBA for 7 weeks for adventitious root induction.

Vigorous, well-rooted plantlets were used for acclimatization. For pre-transplant hardening, tap water (approx. 1.5–2 cm deep) was added to the culture vessels, which were then kept in a shaded environment for 2–3 days. Subsequently, the plantlets were removed from the vessels and residual medium was washed from the roots. The cleaned plantlets were transplanted into plastic pots (10 cm in diameter) filled with a substrate mixture of peat and perlite (1:1, v/v). The potted plantlets were transferred to a greenhouse and covered with transparent plastic film to maintain a high relative humidity of 75–80%. Greenhouse conditions were maintained at a temperature of 25 ± 3 °C with a light intensity of 42 µmol·m^-^²·s^-^¹. After two weeks, the plastic covers were gradually removed to acclimatize the plantlets to the ambient environment. Plantlets were transferred to larger pots as required, and their long-term growth status was periodically recorded.

### 2.6 Axillary bud induction and shoot elongation for culture re-establishment

Re-initiated explants (obtained as described in Section 2.3) were inoculated onto WPM medium to screen for the optimal axillary bud induction protocol. A two-factor experiment was conducted to evaluate the combined effects of activated charcoal (AC; 0 and 2g·L^-^¹) and five specific pairs of 6-BA and NAA. The treatments consisted of 6-BA (0.1, 0.3, 0.5, 0.8, and 1.0 mg·L^-^¹) combined with NAA (0.01, 0.03, 0.05, 0.08, and 0.1 mg·L^-^¹), respectively. Each of the 10 treatments consisted of 30 replicates, with one explant per culture vessel (100 mL flask containing 50 mL of medium). The axillary bud induction rate (%) was determined after 4 weeks. To prevent browning, explants were transferred to fresh medium of the same formulation every 2 weeks.

To optimize shoot elongation, single axillary shoots (hereinafter referred to as re-established shoots) derived from the optimal induction treatment were transferred to fresh WPM medium. A factorial experiment was conducted to test the combined effects of 6-BA (0, 0.1, and 0.2 mg·L^-^¹) and GA_3_ (0.2, 0.5, 0.8, 1.2, and 1.5 mg·L^-^¹). Each of the 15 treatments consisted of 30 replicates. The total culture period was 6 weeks, during which shoots were subcultured every 2 weeks onto fresh medium. At the end of the culture period, the shoot elongation rate (%) and mean shoot length (cm) were recorded.

Following data collection, the elongated shoots were bisected into apical and basal segments to maintain the “*in vitro*–ex vivo–*in vitro*” cycle. The apical segments were subsequently used for adventitious root induction (see Section 2.7), while the basal segments were returned to the optimized axillary bud induction medium to sustain the multiplication cycle.

### 2.7 Rooting and acclimatization of re-established shoots

Elongated shoots (approx. 3–4 cm) obtained from the shoot elongation stage (Section 2.6) were selected for the rooting experiment. A two-factor experiment was conducted to investigate the combined effects of a pulse pre-treatment and IBA concentration in the culture medium.

The shoots were divided into two groups based on the pre-treatment: (1) Pre-treatment group: Shoots were chemically pulse-treated by immersing their basal ends for 30 min in a solution containing 10 mg·L^-^¹ NAA, 20 mg·L^-^¹ IBA, and 10 mg·L^-^¹ putrescine; (2) Control group: Shoots were untreated (dipped in sterile distilled water).

Following the pre-treatment, shoots from both groups were inoculated onto half-strength (½) MS basal medium supplemented with IBA at concentrations of 0, 0.1, 0.3, 0.5, and 0.7 mg·L^-^¹. Each of the 10 treatments consisted of 40 replicates, with one shoot per culture vessel (100 mL flask containing 50 mL of medium). After 8 weeks of culture, the adventitious rooting rate (%) and the mean number of adventitious roots per rooted plantlet were recorded. Vigorous, well-rooted plantlets were subsequently acclimatized and transplanted to the greenhouse following the protocol described in Section 2.5.

### 2.8 Genetic fidelity analysis

Fresh, young leaves (approx. 100 mg) for genomic DNA extraction were collected from three groups: (1) plants derived from cuttings of the wild mother tree; (2) four acclimatized plantlets derived from micrografted primary shoots; and (3) eleven randomly selected acclimatized plantlets derived from re-established shoots. The leaves were flash-frozen in liquid nitrogen and ground to a fine powder. DNA extraction followed a modified Cetyltrimethylammonium Bromide (CTAB) method (Gawel and Jarret, 1991). The powder was lysed in 700 µL of pre-warmed (65 °C) CTAB buffer, incubated for 60 min, and purified with 400 µL of chloroform:isoamyl alcohol (24:1, v/v). DNA was precipitated with ice-cold isopropanol at -20 °C for 2 h. The resulting DNA pellet was washed twice with 75% ethanol, air-dried, and resuspended in 50 µL of sterile water. The concentration and purity of the DNA were determined by measuring the A260/280 ratio using a NanoDrop spectrophotometer (Thermo Fisher Scientific, USA), with ratios between 1.8 and 2.0 considered suitable for PCR analysis.

The specific sequences and characteristics of the primers used in this study are listed in **Supplementary Table 1** (RAPD) and **Supplementary Table 2** (ISSR). PCR amplifications were performed in a 20 µL reaction volume containing: 10 µL of 2× SanTaq PCR Master Mix, 2 µL of primer (10 µM), 2 µL of template DNA (approx. 200 ng), and 6 µL of nuclease-free ddH_2_O. The thermal cycling program for RAPD analysis was: initial denaturation at 94 °C for 5 min; followed by 40 cycles of denaturation at 94 °C for 35 s, annealing at 37 °C for 35 s, and extension at 72 °C for 2 min; with a final extension at 72 °C for 10 min. The thermal cycling program for ISSR analysis was: initial denaturation at 94 °C for 5 min; followed by 40 cycles of denaturation at 95 °C for 40 s, annealing at an optimal temperature (listed in **Supplementary Table 2**) for 35 s, and extension at 72 °C for 2 min; with a final extension at 72 °C for 10 min. The amplification products were separated by electrophoresis on a 1.2% (w/v) agarose gel in 1× TAE buffer, run at 120 V for 30 min. Appropriate DNA size markers, including DL2000 and DL5000 (Vazyme, Nanjing, China), were used to estimate the size of the amplification products. The gels were stained with SafeView™ and visualized under UV transillumination using a gel documentation system (Bio-Rad Gel Doc XR+, USA). The banding patterns of the regenerated plantlets were compared with those of the control plants to assess genetic fidelity.

### 2.9 Statistical analysis

All experiments were arranged in a completely randomized design. The specific sample sizes (replicates) for each experiment are detailed in their respective sections. The propagation parameters were calculated using the following formulas:


$$\text{Contamination Rate }(\%)=\frac{\text{Number of contaminated explants}}{\text{Total number of initial explants}}\times 100\%;$$

$$\text{Survival Rate }(\%)=\frac{\text{Number of surviving explants}}{\text{Total number of initial explants}}\times 100\%;$$

$$\text{Axillary Bud Induction Rate }(\%)=\frac{\text{Number of explants with axillary buds}}{\text{Total number of initial explants}}\times 100\%;$$

$$\text{Callus Formation Rate}\;(\%)=\frac{\text{Number of explants forming callus}}{\text{Total number of initial explants}}\times 100\%;$$

$$\text{Elongation Rate }(\%)=\frac{\text{Number of elongated shoots}}{\text{Number of shoots on elongation medium}}\times 100\%;$$

$$\text{Average Length }(\text{cm})=\frac{\text{Sum of the lengths of all elongated shoots}}{\text{Number of elongated shoots}};$$

$$\begin{array}{c} \text{ Adventitious Rooting Rate }(\%)=\\ \frac{\text{ Number of rooted shoots}}{\text{Total number of shoots transferred to rooting medium}}\times 100\%; \end{array}$$

$$\text{Mean Number of Adventitious Roots}=\frac{\text{Total number of adventitious roots}}{\text{Total number of rooted shoots}};$$

All experimental data were evaluated using one-way analysis of variance (ANOVA). Differences between means were assessed using Tukey’s *post-hoc* multiple comparison test at a significance level of *P* ≤ 0.05. Statistical analyses were performed using SPSS software (IBM SPSS Statistics 27.0). Data are presented as means ± standard deviation (SD).

## 3 Results

### 3.1 Primary culture and bud induction

Following surface sterilization, a total of 45 wild explants were initiated into primary culture. Given the scarcity of source material from the single wild mother tree, extensive optimization of the sterilization protocol was not feasible, resulting in a survival rate of 66.7% (30 viable aseptic explants) after two weeks. These survivors were subsequently subjected to bud induction trials evaluating various 6-BA and NAA combinations (**Table 1**).

**Table 1:** Effect of 6-BA and NAA on axillary bud induction and callus formation of *C. putoensis* explants.

Treatment no.	6-BA (mg·L^-^¹)	NAA (mg·L^-^¹)	Axillary bud induction rate (%)	Callus formation rate (%)	Explant status description
1	0	0	14.3 ± 4.8 ^b^	0.0 ± 0.0 ^c^	Slow bud elongation with occasional callus at the base.
2	0.5	0.05	28.6 ± 6.9 ^a^	14.3 ± 4.8 ^b^	Vigorous axillary bud sprouting with a small amount of green callus.
3	1	0.2	0.0 ± 0.0 ^c^	85.7 ± 9.3 ^a^	Axillary bud growth completely inhibited; profuse callus formation.
4	3	0.5	0.0 ± 0.0 ^c^	100.0 ± 0.0 ^a^	Explants fully covered by callus, which turned brown and died within 4 weeks.

Values are means ± SD. Different lowercase letters within the same column indicate significant differences at *P* < 0.05 according to Tukey’s HSD test.

The morphological responses of the explants exhibited a clear dose-dependent pattern. In the hormone-free control (Treatment 1), axillary bud development was sluggish. Although buds remained viable, they exhibited minimal elongation with only occasional basal callus formation (**Figure 1C**), yielding a significantly low axillary bud induction rate of 14.3 ± 4.8%. Optimal induction was achieved in Treatment 2 (0.5 mg·L^-^¹ 6-BA + 0.05 mg·L^-^¹ NAA). Under these conditions, explants displayed vigorous sprouting characterized by expanded green leaves and minimal basal callus (**Figure 1D**), resulting in the highest axillary bud induction rate of 28.6 ± 6.9%.

Conversely, elevated concentrations of plant growth regulators (PGRs) shifted the developmental trajectory from organogenesis to callogenesis. In Treatment 3 (1.0 mg·L^-^¹ 6-BA + 0.2 mg·L^-^¹ NAA), axillary bud growth was completely inhibited in favor of profuse callus formation (85.7 ± 9.3%; **Figure 1E**), with zero shoot regeneration observed. At the highest concentrations tested (Treatment 4; 3.0 mg·L^-^¹ 6-BA + 0.5 mg·L^-^¹ NAA), excessive hormonal levels proved detrimental. While the callus formation rate reached 100%, the explants were entirely enveloped by non-regenerative callus that rapidly became necrotic within four weeks (**Figure 1F**). These results indicate that high PGR concentrations are toxic to wild *C. putoensis* explants, identifying the low-concentration combination in Treatment 2 as the only viable strategy for establishing primary cultures.

### 3.2 Micrografting-assisted rooting of primary shoots

To overcome the rooting recalcitrance of primary shoots, a heterologous Hypocotyl-Apical Bud (HAB) micrografting technique was utilized. Healthy primary shoots (**Figure 2A**) were prepared as scions (**Figure 2B**), while soybean (*Glycine max*) seedlings were germinated (**Figure 2C**) to provide excised hypocotyls as nurse rootstocks (**Figure 2D**).

During co-culture, the graft union exhibited distinct morphological changes. Initial callus proliferation was observed at the junction by day 5 (**Figure 2E**). By day 10, the basal ends of the *C. putoensis* scions became noticeably swollen (**Figure 2F**), signaling the successful initiation of adventitious root primordia.

Following the two-week induction phase, the scions were excised from the rootstocks and transferred to WPM rooting medium. Within seven weeks, a robust root system was established (**Figure 2G**). The resulting plantlets were successfully hardened and acclimatized, maintaining vigorous growth for 2.5 years ex vitro (**Figure 2H**). These plantlets subsequently served as rejuvenated source materials for culture re-establishment.

### 3.3 Surface sterilization and re-initiation of acclimatized regenerants

Twelve treatments were assessed to optimize the surface sterilization protocol for re-initiating acclimatized *C. putoensis* regenerants (**Table 2**). Among these, Treatment 11 (75% ethanol for 50 s, followed by 50% PPM for 7 min) achieved the most effective balance between decontamination and explant viability, yielding a low contamination rate of 5.00% and the highest survival rate of 92.78%.

**Table 2:** Effect of surface sterilization treatments on contamination and survival rates of *C. putoensis* explants.

Treatment no.	Ethanol treatment (s)	PPM stock conc. (%)	Mixture treatment (min)	Contamination rate (%)	Survival rate (%)
1	30	20	5	78.33 ± 3.51 ^f^	15.56 ± 2.40 ^f^
2	30	20	7	65.00 ± 2.89 ^e^	28.89 ± 3.11 ^e^
3	30	20	9	51.67 ± 3.14 ^d^	42.22 ± 2.95 ^d^
4	30	50	5	38.89 ± 2.54 ^c^	55.56 ± 3.40 ^c^
5	30	50	7	25.00 ± 2.08 ^b^	72.22 ± 2.88 ^b^
6	30	50	9	18.33 ± 1.57 ^ab^	78.89 ± 2.15 ^b^
7	50	20	5	35.56 ± 2.15 ^c^	59.44 ± 3.01 ^c^
8	50	20	7	22.22 ± 1.88 ^b^	75.00 ± 2.50 ^b^
9	50	20	9	16.67 ± 1.48 ^ab^	81.11 ± 1.96 ^ab^
10	50	50	5	14.44 ± 1.29 ^ab^	85.56 ± 1.88 ^a^
11	50	50	7	5.00 ± 0.22 ^a^	92.78 ± 0.26 ^a^
12	50	50	9	2.22 ± 0.48 ^a^	45.00 ± 3.85 ^d^

Values are means ± SD. Different lowercase letters within the same column indicate significant differences at *P* < 0.05 according to Tukey’s HSD test.

The results indicated that both ethanol immersion time and PPM concentration significantly influenced sterilization efficiency. Increasing the ethanol duration from 30 s to 50 s substantially improved the survival of aseptic explants by suppressing latent contaminants; for instance, Treatment 8 (50 s) achieved a 75.00% survival rate, whereas Treatment 2 (30 s) yielded only 28.89%. Similarly, 50% PPM was more effective than 20% PPM in reducing microbial outbreaks. However, prolonged exposure to the sterilants induced severe phytotoxicity. Although Treatment 12 (9 min) achieved the lowest contamination rate (2.22%), it caused significant tissue damage, reducing the survival rate to 45.00%. Due to its high efficiency and minimal toxicity, Treatment 11 was selected for the cyclic micropropagation system.

### 3.4 Axillary bud induction during culture re-establishment

The synergistic effects of 6-BA, NAA, and activated charcoal (AC) on the axillary bud induction during culture re-establishment were evaluated (**Table 3**). Compared to the recalcitrant primary explants, these shoots—derived from rejuvenated greenhouse regenerants—exhibited a significantly more robust morphogenetic response. The highest induction rate (90.31%) was achieved in Treatment 7 (0.3 mg·L^-^¹ 6-BA + 0.03 mg·L^-^¹ NAA + 2 g·L^-^¹ AC), which was identified as the optimal formulation for germplasm expansion.

**Table 3:** Effect of activated charcoal (AC) and hormones on axillary bud induction of *C. putoensis*.

Treatment no.	AC (g·L^-^¹)	6-BA (mg·L^-^¹)	NAA (mg·L^-^¹)	Axillary bud induction rate (%)
1	0	0.1	0.01	65.46 ± 4.53 ^f^
2	0	0.3	0.03	82.58 ± 3.81 ^c^
3	0	0.5	0.05	80.20 ± 4.15 ^cd^
4	0	0.8	0.08	74.11 ± 5.18 ^e^
5	0	1	0.1	63.37 ± 5.52 ^f^
6	2	0.1	0.01	71.84 ± 4.24 ^e^
7	2	0.3	0.03	90.31 ± 3.54 ^a^
8	2	0.5	0.05	86.52 ± 3.92 ^b^
9	2	0.8	0.08	78.51 ± 4.87 ^d^
10	2	1	0.1	68.13 ± 5.33 ^f^

Values are means ± SD. Different lowercase letters within the same column indicate significant differences at *P* < 0.05 according to Tukey’s HSD test.

The addition of AC consistently promoted axillary budding in the re-established nodal segments. At the optimal PGR concentration, Treatment 7 (with 2 g·L^-^¹ AC) significantly enhanced the induction rate compared to its AC-free counterpart (82.58%; Treatment 2). Furthermore, induction efficiency followed a dose-dependent pattern, peaking at moderate PGR levels (0.3 mg·L^-^¹ 6-BA and 0.03 mg·L^-^¹ NAA) but declining under supra-optimal concentrations. This suggests a specific hormonal sensitivity threshold in the rejuvenated tissues.

**Figure 3:**
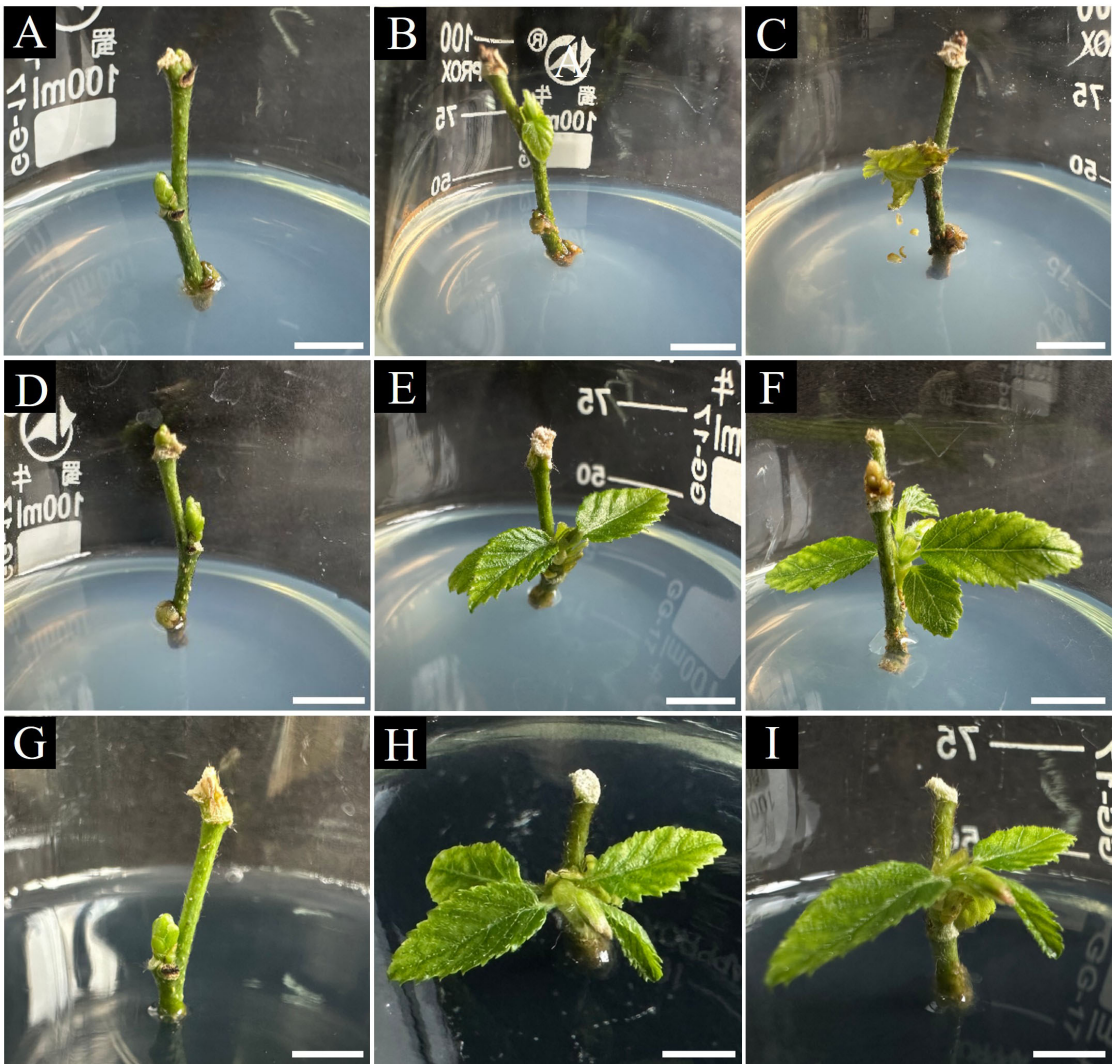
Effects of hormone concentrations and activated charcoal on *C. putoensis* axillary bud induction. **(A–C)** Explants on WPM medium supplemented with 0.8 mg·L^-^¹ 6-BA and 0.08 mg·L^-^¹ NAA at 1 week **(A)**, 3 weeks **(B)**, and 4 weeks **(C)**. **(D–F)** Explants on WPM medium supplemented with 0.3 mg·L^-^¹ 6-BA and 0.03 mg·L^-^¹ NAA at 1 week **(D)**, 3 weeks **(E)**, and 4 weeks **(F)**. **(G–I)** Explants on the same medium as **(D–F)** with 2 g·L^-^¹ activated charcoal (AC) at 1 week **(G)**, 3 weeks **(H)**, and 4 weeks **(I)**. Scale bars: **(A, F)** = 0.9 cm; **(G)** = 1.0 cm; **(B–D, H, I)** = 1.1 cm; **(E)** = 1.3 cm.

Morphological observations corroborated these quantitative findings. Shoots in Treatment 7 displayed vigorous growth, characterized by accelerated internode elongation and healthy leaf expansion (**Figures 3G–I**). Conversely, AC-free shoots (Treatment 2) appeared less vigorous (**Figures 3D–F**), while those exposed to excessive PGR concentrations exhibited stunted growth and reduced vitality (**Figures 3A–C**), highlighting the necessity of precise hormonal calibration.

### 3.5 Shoot elongation during culture re-establishment

The elongation of re-established shoots was significantly influenced by the synergy between 6-BA and GA_3_ over the 6-week culture period (**Table 4**). Compared to the hormone-free controls (Treatments 1–5), the supplementation of 0.1 mg·L^-^¹ 6-BA substantially enhanced both elongation rates and mean shoot length. Within the 0.1 mg·L^-^¹ 6-BA series, GA_3_ exhibited a distinct dose-dependent effect, with growth parameters peaking at 0.8 mg·L^-^¹.

**Table 4:** Effect of 6-BA and GA_3_ on shoot elongation of *C. putoensis*.

Treatment no.	6-BA (mg·L^-^¹)	GA_3_ (mg·L^-^¹)	Elongation rate (%)	Average length (cm)
1	0	0.2	52.17 ± 5.11 ^g^	1.12 ± 0.34 ^h^
2	0	0.5	60.39 ± 4.18 ^f^	1.54 ± 0.42 ^g^
3	0	0.8	67.25 ± 3.53 ^e^	1.82 ± 0.41 ^f^
4	0	1.2	64.18 ± 4.82 ^ef^	2.14 ± 0.53 ^e^
5	0	1.5	57.42 ± 5.49 ^fg^	2.05 ± 0.58 ^e^
6	0.1	0.2	70.47 ± 4.12 ^de^	1.73 ± 0.44 ^f^
7	0.1	0.5	81.33 ± 3.33 ^ab^	3.21 ± 0.51 ^c^
8	0.1	0.8	88.16 ± 2.84 ^a^	4.53 ± 0.78 ^a^
9	0.1	1.2	85.65 ± 3.11 ^a^	4.38 ± 0.72 ^a^
10	0.1	1.5	78.19 ± 3.94 ^bc^	4.06 ± 0.63 ^ab^
11	0.2	0.2	65.38 ± 4.21 ^ef^	1.59 ± 0.38 ^g^
12	0.2	0.5	76.51 ± 3.88 ^bc^	2.88 ± 0.47 ^d^
13	0.2	0.8	82.15 ± 3.14 ^ab^	3.95 ± 0.53 ^b^
14	0.2	1.2	79.44 ± 2.97 ^b^	3.67 ± 0.59 ^bc^
15	0.2	1.5	71.82 ± 3.56 ^d^	3.15 ± 0.61 ^c^

Values are means ± SD. Different lowercase letters within the same column indicate significant differences at *P* < 0.05 according to Tukey’s HSD test.

**Figure 4:**
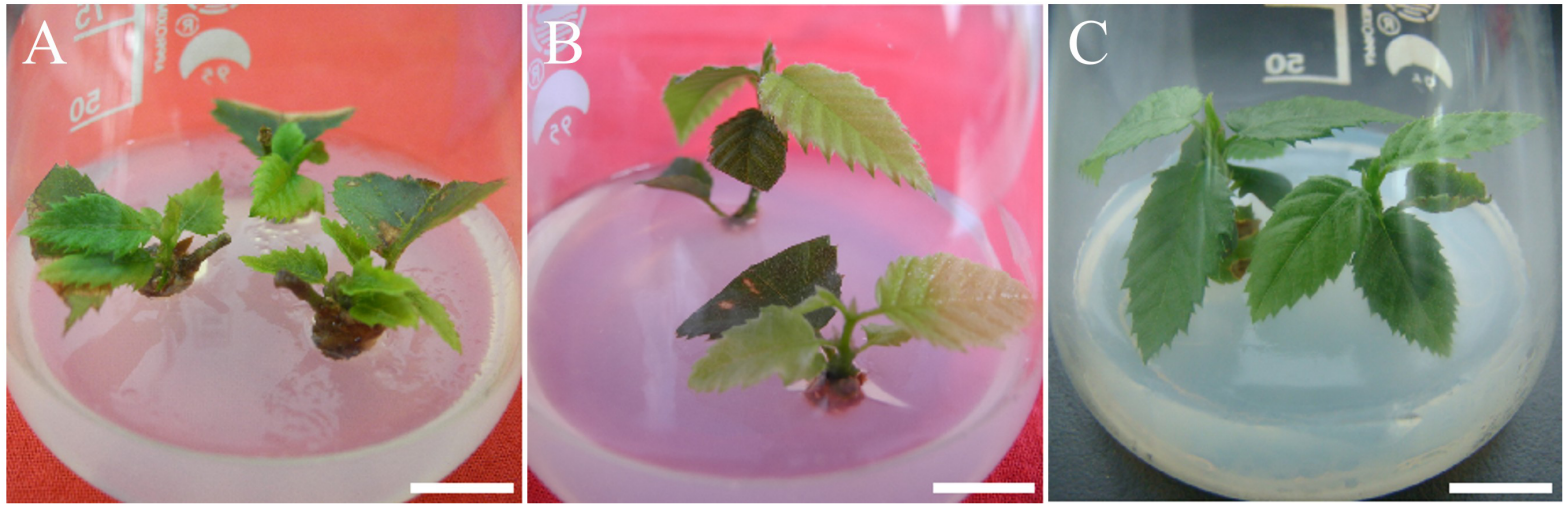
Shoot elongation of re-established *C. putoensis* on optimal medium. **(A–C)** Shoots on WPM medium supplemented with 0.1 mg·L^-^¹ 6-BA and 0.8 mg·L^-^¹ GA_3_ at 2 weeks **(A)**, 3 weeks **(B)**, and 6 weeks **(C)**. Scale bars: **(A, C)** = 1.1 cm; **(B)** = 1 cm.

Consequently, the maximum elongation rate (88.16 ± 2.84%) and mean shoot length (4.53 ± 0.78 cm) were achieved in Treatment 8 (0.1 mg·L^-^¹ 6-BA + 0.8 mg·L^-^¹ GA_3_). Under these optimized conditions, the shoots displayed vigorous vegetative growth, with significant elongation recorded at 2, 3, and 6 weeks (**Figures 4A–C**). Neither increasing GA_3_ to 1.2 mg·L^-^¹ nor 6-BA to 0.2 mg·L^-^¹ yielded further statistical benefits, identifying the combination in Treatment 8 as the optimal synergistic threshold. These robust shoots sustained the cyclic propagation system: apical segments were advanced to the rooting phase, while basal segments were returned to axillary bud induction, ensuring continuous and efficient production of rejuvenated *C. putoensis* germplasm.

### 3.6 Rooting and acclimatization of re-established shoots

**Figure 5:**
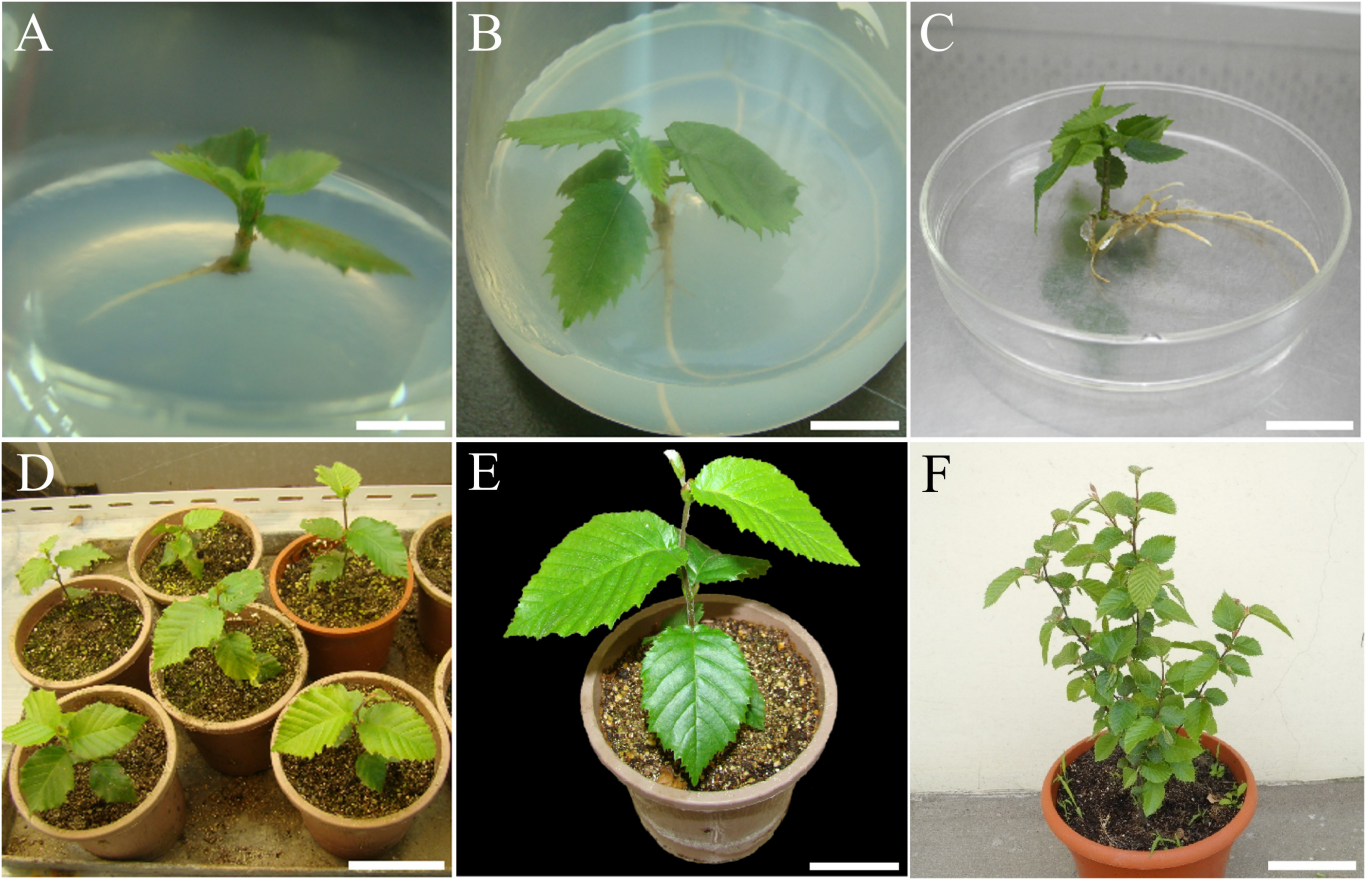
Rooting and acclimatization of re-established *C. putoensis*. **(A)** Adventitious rooting on MS medium with 0.3 mg·L^-^¹ IBA after 7 weeks (no pre-treatment). **(B)** Adventitious rooting on same medium after 7 weeks (with pre-treatment). **(C)** Root system of pre-treated plantlet after 8 weeks. **(D)** Acclimatized plantlets after 50 days ex vitro growth. **(E)** Single acclimatized plantlet after 50 days. **(F)** Plant after 2 years ex vitro growth (Source for subsequent cycles). Scale bars: **(A)** = 0.8 cm; **(B)** = 1.1 cm; **(C)** = 2.3 cm; **(D)** = 9 cm; **(E)** = 4.3 cm; **(F)** = 11 cm.

Adventitious rooting in re-established shoots was significantly enhanced by a synergistic protocol of chemical pre-treatment and subsequent auxin stimulation (**Table 5**). While untreated explants exhibited spontaneous rooting, the NAA/IBA/putrescine pre-treatment was essential for high-frequency induction, markedly increasing both rooting rate and root number. The optimal response (89.33 ± 3.42% rooting; 3.11 ± 0.45 roots/plantlet) was achieved in Treatment 3 (0.3 mg·L^-^¹ IBA), substantially outperforming the best untreated control (52.64%; Treatment 8) (**Figures 5A, B**).

**Table 5:** Effect of pre-treatment and IBA on adventitious rooting of *C. putoensis* shoots.

Treatment no.	Pre-treatment	Concentration of IBA (mg·L^-^¹)	Adventitious rooting rate (%)	Mean number of adventitious roots per rooted shoot
1	+	0	0.00 ± 0.00 ^g^	–
2	+	0.1	78.24 ± 4.15 ^b^	2.41 ± 0.38 ^bc^
3	+	0.3	89.33 ± 3.42 ^a^	3.11 ± 0.45 ^a^
4	+	0.5	83.71 ± 3.96 ^ab^	2.87 ± 0.41 ^ab^
5	+	0.7	75.00 ± 4.50 ^b^	1.56 ± 0.33 ^d^
6	–	0	0.00 ± 0.00 ^g^	–
7	–	0.1	41.55 ± 5.23 ^e^	1.85 ± 0.37 ^cd^
8	–	0.3	52.64 ± 4.87 ^c^	2.68 ± 0.52 ^b^
9	–	0.5	48.18 ± 5.12 ^d^	2.23 ± 0.44 ^c^
10	–	0.7	35.50 ± 4.78 ^f^	1.15 ± 0.29 ^e^

The “+” indicates shoots subjected to a rooting pre-treatment, while “-” indicates the untreated control group. Values are means ± SD. Different lowercase letters within the same column indicate significant differences at *P* < 0.05 according to Tukey’s HSD test.

Both groups displayed a dose-dependent response to IBA, with performance peaking at 0.3 mg·L^-^¹ and declining under supra-optimal levels (0.5–0.7 mg·L^-^¹). The total failure of rooting in hormone-free media (0.00%) confirms that exogenous auxin is indispensable for initiating rhizogenesis in this species. By week 8, plantlets with vigorous, well-branched root systems (**Figure 5C**) were successfully acclimatized, maintaining morphological fidelity to the wild mother tree (**Figures 5D–F**).

### 3.7 Genetic fidelity assessment of regenerated plantlets

**Figure 6:**
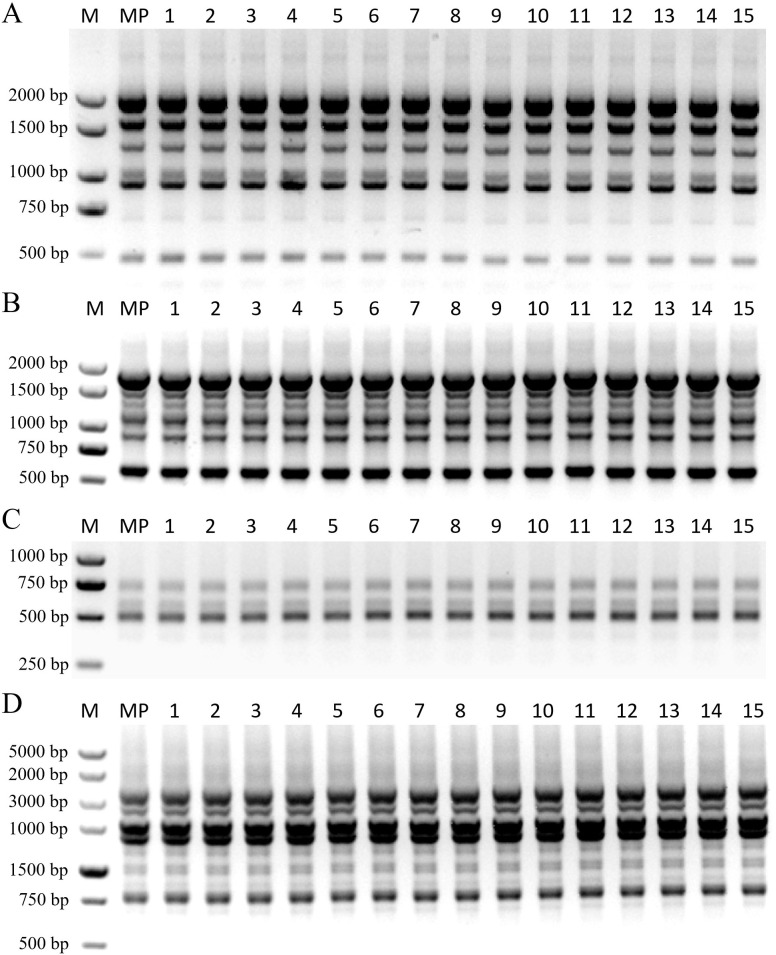
Genetic fidelity of regenerated *C. putoensis* using RAPD and ISSR markers. **(A, B)** RAPD profiles with primers S181 **(A)** and S49 **(B)**. **(C, D)** ISSR profiles with primers UBC851 **(C)** and UBC881 **(D)**. Lanes: M, DNA molecular weight marker [2000 bp for **(A, B)**; 1000 bp for **(C)**; 5000 bp for **(D)**]; MP, mother tree cutting (Control); 1–4, plantlets from primary culture; 5–15, plantlets from re-established culture.

Genetic fidelity was assessed using 19 RAPD and 24 ISSR primers, generating 158 and 125 clear bands, respectively, for a total of 283 scorable loci. Representative primers, including RAPD (S181, S49) and ISSR (UBC851, UBC881), produced distinct and reproducible banding patterns (**Figures 6A–D**). Across all assays, amplification profiles were uniformly monomorphic among the cutting-derived mother plant (MP), the four plantlets from the primary culture (Lanes 1–4), and the eleven from the re-established pathway (Lanes 5–15). As illustrated in **Figure 6**, all samples exhibited identical banding patterns with no polymorphic loci detected. These results confirm that the cyclic micropropagation system preserves high genetic fidelity to the donor genotype, ensuring that regenerated *C. putoensis* plantlets remain true-to-type without undergoing somaclonal variation.

## 4 Discussion

*C. putoensis*, a critically endangered species with inherent reproductive limitations, urgently requires artificial propagation for conservation (Li et al., 2010). Previous research focused on distribution (He, 2009), chloroplast genomics (Yoo and Wen, 2007), karyology (Yang et al., 2020), and ecophysiological responses (Sheng et al., 2021b), leaving a gap for a complete micropropagation system validating genetic fidelity throughout the “*in vitro*–ex vivo–*in vitro*” cycle. This study establishes a comprehensive cyclic regeneration system by integrating effective sterilization for recalcitrant explants, micrografting to overcome rooting barriers, a stable re-established culture loop, and an optimized two-step rooting protocol. Validation via RAPD and ISSR markers confirmed 100% genetic fidelity in regenerants, providing a robust technical foundation for the *ex situ* conservation of *C. putoensis*.

### 4.1 Challenges in primary culture

The differential response of wild explants to hormones highlighted limitations in organogenic potential. While low 6-BA/NAA concentrations induced axillary bud sprouting with high genetic fidelity (**Table 1**), the efficiency (28.6%) was insufficient for rapid multiplication. Conversely, high hormone concentrations aiming for adventitious bud induction proved ineffective; the resulting callus was non-regenerative and necrotic (**Table 1**; **Figures 1E, F**), indicating severe recalcitrance toward indirect organogenesis. This may stem from imbalanced endogenous hormones, where high exogenous levels trigger cell death rather than differentiation (Hu et al., 2017). Given the infeasibility of the callus pathway and the inefficiency of axillary budding alone, rooting primary shoots via micrografting became the essential bridge to establish sustainable ex vivo donor plants.

### 4.2 Physiological mechanisms of micrografting-mediated rescue

The critical rooting bottleneck in primary shoots was overcome by the HAB (Hypocotyl-Apical Bud) micrografting system. While heterologous micrografting aids other recalcitrant species (Wang et al., 2022), the specific selection of the rootstock is decisive for graft compatibility and success. In this study, soybean (*Glycine max* cv. ‘Jiaoda 18’) was selected from candidate species due to its distinct physiological and morphological advantages identified in preliminary screenings. Physiologically, this cultivar exhibits high endogenous auxin levels, evidenced by spontaneous rooting capabilities, which compensates for the hormonal deficiencies in the recalcitrant *C. putoensis* scion. Morphologically, the hypocotyl diameter of soybean can be precisely regulated by 6-BA treatment (as described in Methods) to perfectly match the *C. putoensis* scion, ensuring optimal vascular alignment and mechanical stability. This successful combination likely results from rootstock-scion interactions (Rasool et al., 2020): the physiologically active soybean rootstock establishes favorable hormonal gradients (Sharma and Zheng, 2019) and supply mobile signaling molecules and carbohydrates deficient in the scion (Druege et al., 2019; Lebedeva et al., 2020; Deng et al., 2021). Furthermore, the graft interface wound response might synergize with rootstock signals to activate rhizogenesis regulators like the WOX11/LBD module (Liu et al., 2014; Iwase et al., 2021; Wan et al., 2023). Concurrently, the rootstock likely acts as a sink to detoxify rooting inhibitors (De Klerk et al., 2011; Rasool et al., 2020). Thus, the HAB system served as a physiological bridge to generate robust acclimatized plants (**Figure 2G**).

### 4.3 Optimization of the re-established culture system

Establishing a re-established culture system from acclimatized plantlets enabled scalable propagation. We utilized WPM medium, suitable for woody plant culture due to lower salt concentrations (Lloyd and McCown, 1980). Moderate 6-BA/NAA concentrations supplemented with activated charcoal (AC) proved optimal for axillary bud induction (**Table 3**), suggesting a requirement for precise hormonal balance to activate response regulators like B-ARRs (Su et al., 2011). AC significantly enhanced induction (**Figures 3G–I**), likely by adsorbing inhibitory phenolics (Thomas, 2008). For shoot elongation, a synergy between low-concentration 6-BA and GA_3_ was observed (**Table 4**); while GA_3_ drives elongation via the GA-GID1-DELLA pathway (Sun, 2011), trace 6-BA may maintain physiological competence or enhance GA_3_ responsiveness (Hartmann et al., 2011; Müller and Leyser, 2011) without inhibiting apical growth (Weiss and Ori, 2007). This stage-specific optimization established an efficient multiplication protocol for re-established shoots (**Figures 4A–C**).

### 4.4 Enhanced rooting competence in re-established shoots

The re-established culture significantly improved rooting competence, likely due to physiological rejuvenation enhancing auxin responsiveness (Wang et al., 2023). The optimized two-step protocol—a high-concentration hormonal pre-treatment followed by culture on half-strength medium with 0.3 mg·L^-^¹ IBA—proved highly effective (**Table 5**). The initial pulse functions as an inductive trigger for rhizogenesis (De Klerk et al., 1999), while the subsequent low-salt/low-hormone environment supports root development without toxicity or excessive callusing (Castiglione et al., 1993; Bai et al., 2020). Achieving an 89.33% rooting rate (vs. 52.64% in controls; **Figures 5A, B**), this protocol marks a successful transition from micrografting reliance to a standardized chemical induction method suitable for mass propagation.

### 4.5 Genetic stability and conservation implications

Maintaining genetic integrity is critical for *C. putoensis* due to its narrow genetic base, where somaclonal variation poses a risk of irreversible genotype loss (Krishna et al., 2016; Duta-Cornescu et al., 2023). Our dual-marker validation confirms the system’s reliability; this high fidelity is attributed to the exclusive use of the pre-existing meristem-based axillary pathway, which is inherently more stable than adventitious organogenesis (Ngezahayo and Liu, 2014; Krishna et al., 2016). This provides essential quality assurance for *ex situ* conservation. While this study established a functional cyclic system, limitations regarding wild resource scarcity and seasonality persist. Future research should focus on broadening propagation strategies, such as somatic embryogenesis, and investigating the molecular mechanisms governing morphogenesis to facilitate large-scale multiplication.

## 5 Conclusions

**Figure 7:**
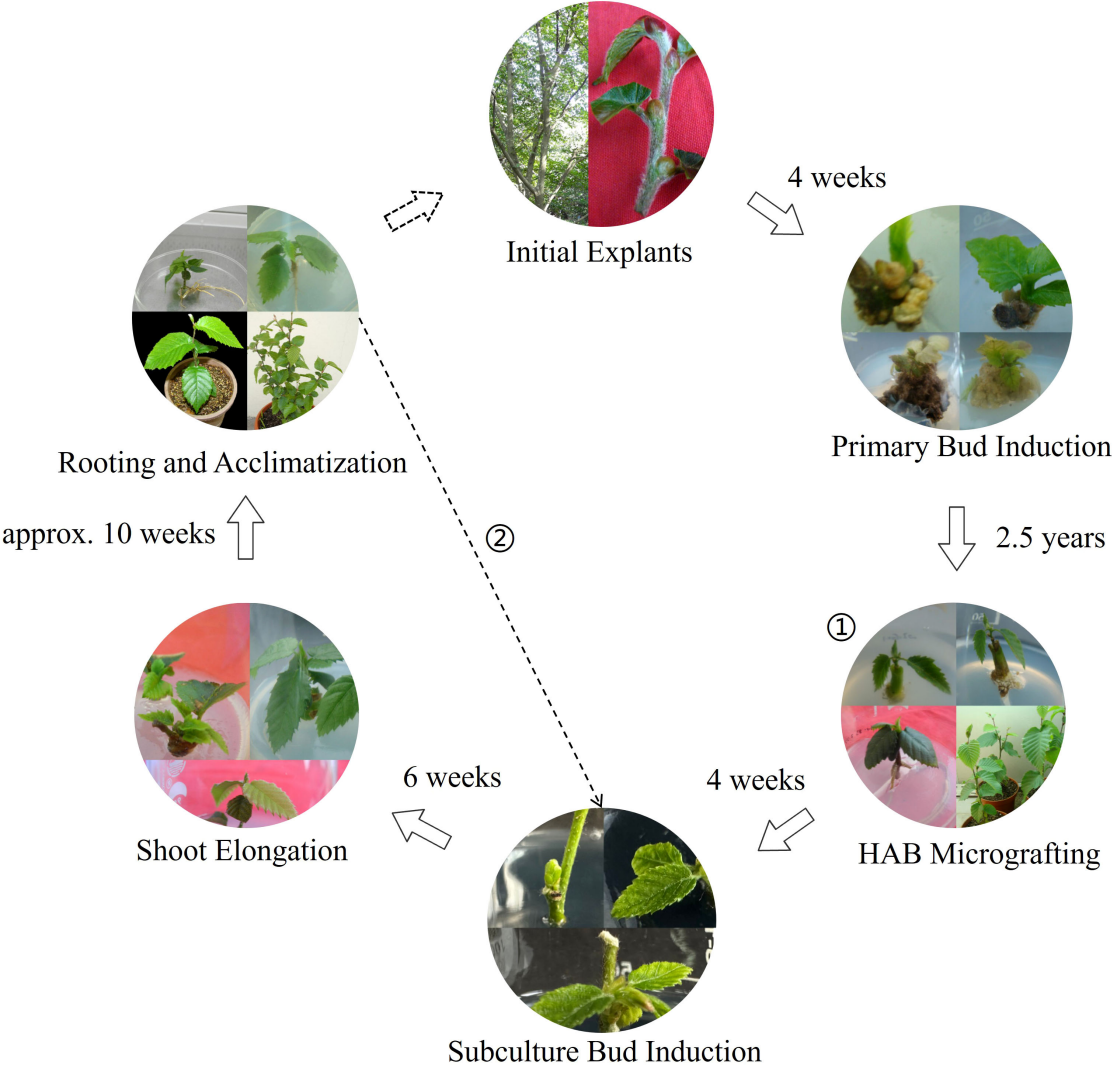
Flow scheme of the cyclic *in vitro* regeneration system for *C. putoensis*. The diagram illustrates the six stages of the “*in vitro*–ex vivo–*in vitro*” cycle with approximate durations. Note: The dashed arrow signifies the transition from initial establishment to a self-sustaining system; once the re-established culture loop is operational, dependency on wild source material (Initial Explants) is eliminated. The symbol ① denotes acclimatized plantlets from the primary HAB micrografting pathway, serving as the ex vivo donor stock for initiating the re-established culture. The symbol ② represents regenerated plants from the re-established pathway, serving as an explant source independent of the wild mother plant.

This study successfully establishes the first complete, efficient, cyclic, and genetically stable micropropagation system for the critically endangered *C. putoensis* (**Figure 7**). By optimizing the sterilization of recalcitrant explants, overcoming primary shoot rooting using micrografting, establishing an efficient subculture cycle based on acclimatized plantlets (including optimized axillary bud induction, elongation, and a two-step rooting method), and ultimately confirming 100% genetic fidelity with molecular markers, this system successfully overcomes the conservation bottlenecks of low propagation efficiency and reliance on a single mother plant. This work not only provides crucial technical support and a reliable source of plant material for the *ex situ* conservation and future population recovery of *C. putoensis*, but its strategies for addressing a series of technical challenges also offer an important methodological reference for the rapid *in vitro* propagation and conservation of other rare, endangered, and particularly recalcitrant woody species.

## Data Availability

The original contributions presented in the study are included in the article/**Supplementary Material**. Further inquiries can be directed to the corresponding authors.
