# Supplementary material for: A novel in vitro cyclic micropropagation protocol and assessment of genetic fidelity in the critically endangered woody species Carpinus putoensis
Source: Front Plant Sci. 2026 Mar 11;17:1791775. doi: 10.3389/fpls.2026.1791775 (PMC13013394; doi:10.3389/fpls.2026.1791775)
Supplement: Supplementary file 1 [file Table1.docx]

Supplementary Material

**Table S1.** Characteristics of RAPD primers used for genetic fidelity analysis of *C. putoensis*.

| No. | Primer Name | Sequence (5'-3') | Number of  Amplified Bands | Band Size Range (bp) |
| --- | --- | --- | --- | --- |
| 1 | S25 | TGCGCCCTTC | 5 | 500 – 1800 |
| 2 | S49 | ACCTGAACGG | 6 | 500 – 1800 |
| 3 | S30 | CTGCTGGGAC | 7 | 300 – 1100 |
| 4 | S31 | TGTCATCCCC | 10 | 250 – 1500 |
| 5 | S130 | AGGGCCGTCT | 13 | 250 – 1800 |
| 6 | S132 | CAGCTCACGA | 9 | 500 – 1700 |
| 7 | S133 | CTCTCCGCCA | 8 | 300 – 1400 |
| 8 | S136 | AGCGTCCTCC | 7 | 250 – 1400 |
| 9 | S144 | GGAAGTCGCC | 9 | 350 – 1000 |
| 10 | S169 | TGGAGAGCAG | 11 | 100 – 1800 |
| 11 | S172 | AGAGGGCACA | 11 | 300 – 2000 |
| 12 | S175 | TCATCCGAGG | 7 | 600 – 2000 |
| 13 | S181 | CTACTGCGCT | 7 | 400 – 2000 |
| 14 | S185 | TTTGGGGCCT | 9 | 350 – 2100 |
| 15 | S205 | GGGTTTGGCA | 8 | 400 – 1300 |
| 16 | S208 | AACGGCGACA | 11 | 250 – 1800 |
| 17 | S221 | TGACGCATGG | 7 | 400 – 1600 |
| 18 | S226 | ACGCCCAGGT | 7 | 550 – 2000 |
| 19 | S230 | GGACCTGCTG | 6 | 450 – 2000 |
| Total | |  | 158 |  |

**Note:** *C. putoensis*, *Carpinus putoensis*; RAPD, random amplified polymorphic DNA. DNA band sizes were estimated relative to the DL2000 DNA ladder. All amplified fragments showed 100% monomorphism between the mother plant and regenerants.

**Table S2.** Characteristics of ISSR primers used for genetic fidelity analysis of *C. putoensis*.

| No. | Primer Name | Sequence (5'-3') | Number of Amplified Bands | Band Size Range (bp) |
| --- | --- | --- | --- | --- |
| 1 | UBC817 | CACACACACACACACAA | 5 | 300 – 1000 |
| 2 | UBC818 | CACACACACACACACAG | 5 | 500 – 1500 |
| 3 | UBC823 | TGTGTGTGTGTGTGTGC | 2 | 350 – 500 |
| 4 | UBC824 | TCTCTCTCTCTCTCTCG | 4 | 500 – 2000 |
| 5 | UBC825 | ACACACACACACACACT | 6 | 350 – 1200 |
| 6 | UBC826 | ACACACACACACACACC | 7 | 550 – 1500 |
| 7 | UBC827 | ACACACACACACACACG | 7 | 550 – 1500 |
| 8 | UBC835 | CTCTCTCTCTCTCTCTYC | 8 | 400 – 2500 |
| 9 | UBC836 | AGAGAGAGAGAGAGAGYA | 4 | 500 – 900 |
| 10 | UBC840 | GAGAGAGAGAGAGAGAYT | 6 | 350 – 1500 |
| 11 | UBC842 | GAGAGAGAGAGAGAGAYG | 3 | 350 – 1000 |
| 12 | UBC844 | CTCTCTCTCTCTCTCTRC | 1 | ~1200 |
| 13 | UBC846 | CACACACACACACACART | 6 | 350 – 700 |
| 14 | UBC851 | CTCTCTCTCTCTCTCTYG | 3 | 500 – 750 |
| 15 | UBC856 | ACACACACACACACACYA | 1 | ~850 |
| 16 | UBC859 | TGTGTGTGTGTGTGTGRC | 5 | 450 – 1800 |
| 17 | UBC860 | TGTGTGTGTGTGTGTGRA | 8 | 400 – 2800 |
| 18 | UBC866 | CTCCTCCTCCTCCTCCTC | 4 | 500 – 2000 |
| 19 | UBC868 | GAAGAAGAAGAAGAAGAA | 3 | 600 – 700 |
| 20 | UBC874 | CCCTCCCTCCCTCCCT | 9 | 1000 – 3200 |
| 21 | UBC880 | GGAGAGGAGAGGAGA | 6 | 500 – 1200 |
| 22 | UBC881 | GGGTGGGGTGGGGTG | 10 | 750 – 2500 |
| 23 | UBC892 | TAGATCTGATATCTGAATTCCC | 3 | 1200 – 2500 |
| 24 | UBC895 | AGAGTTGGTAGCTCTTGATC | 9 | 900 – 4500 |
| Total | |  | 125 |  |

**Note:** *C. putoensis*, *Carpinus putoensis*; ISSR, inter-simple sequence repeat. Band size ranges were estimated relative to the DL2000 and DL5000 DNA ladders. All amplified bands showed 100% monomorphism.
